# Columbianadin attenuates doxorubicin-induced cardiac injury, oxidative stress, and apoptosis via Sirt1/FOXO1 signaling pathway

**DOI:** 10.1590/acb382223

**Published:** 2023-06-26

**Authors:** Bo Peng, Li Rao, Jiaolong Yang, Xiaowei Ku, Bin Kong, Wei Shuai, He Huang

**Affiliations:** 1 Wuhan University – Renmin Hospital – Department of Cardiology – Hubei, China.; 2 Wuhan University – Cardiovascular Research Institute – Hubei, China.; 3 Hubei Key Laboratory of Cardiology – Hubei, China.; 4 Wuhan University – Renmin Hospital – Department of Geriatrics – Hubei, China.; 5 Wuhan University – Renmin Hospital – Department of Neurology – Hubei, China.; 6 Wuhan University – Renmin Hospital – Department of Endocrinology – Hubei, China.

**Keywords:** Doxorubicin, Cardiotoxicity, Oxidative Stress, Apoptosis, Sirtuin 1

## Abstract

**Purpose:**

Oxidative stress and apoptosis contribute to the pathological basis of doxorubicin (DOX)-induced cardiotoxicity. Columbianadin (CBN) is one of the main bioactive constituents isolated from the root of *Angelica pubescens*. Herein, we intended to explore the potential role and molecular basis of CBN in DOX-induced cardiotoxicity.

**Methods:**

C57BL/6 mice were subjected to DOX (15 mg/kg/day, i.p.) to generate DOX-induced cardiotoxicity. CBN (10 mg/kg/day, i.p.) was administered for four week following DOX injection.

**Results:**

DOX administered markedly dampened cardiac function, increased cardiac injury, excessive reactive oxygen species (ROS) production, and cardiomyocyte loss. These alterations induced by DOX significantly alleviated by CBN treatment. Mechanistically, our results demonstrated that the CBN exerts cardioprotection role against DOX by up-regulating silent information regulator 1 (Sirt1) and decreasing acetylation of forkhead box O1 (FOXO1). Moreover, Sirt1 inhibition with Ex-527 significantly blunt the beneficial effect of CBN on DOX-induced cardiotoxicity, including cardiac dysfunction, ROS, and apoptosis.

**Conclusion:**

Collectively, CBN attenuated oxidative stress and cardiomyocyte apoptosis in DOX-induced cardiotoxicity through maintaining Sirt1/FOXO1 signaling pathway. Our results demonstrated that CBN might be used to treat DOX-related cardiotoxicity.

## Introduction

Doxorubicin (DOX), a potent anthracycline, is widely utilized as the first-line drugs to treat human neoplasms, including leukemias, lymphomas and solid malignancies for several decades, but it’s clinically limited due to its cardiotoxicity^1^^,^ 2. Although a lot of molecular mechanisms underlying DOX cardiotoxicity effects have been reported, including iron regulatory protein, nitric oxide release, mitochondrial dysfunction, impaired adenosine triphosphate level, hampered cardiac progenitor cells, calcium dysregulation, inflammatory mediators, endothelial dysfunction, activation of ubiquitin protease system and autophagy, the exact mechanisms is indistinct^3^. Recently, accumulating researches identified the formation of reactive oxygen species (ROS) associated with DOX-related cardiac injury. Excessive ROS could cause harm to cardiomyocyte, lead to cell loss, ultimately heart failure^4^^,^ 5. Hence, finding drugs to curb ROS and cardiomyocyte apoptosis may be beneficial to the therapy of DOX-induced cardiac injury.

Columbianadin (CBN) is one of the main bioactive constituents isolated from the root of *Angelica pubescens* 6, and it is involved in several biological processes^7^^,^ 8. Previous research indicated CBN could inhibit the strength of voltage-gated Ca^2+^ currents in dorsal root ganglion neurons derived from mice^9^. Furthermore, CBN can also decreased peak or late component of voltage-gated Na^+^ current (*I*_Na_) in electrically excitable cells^10^. Recently, CBN was reported to play role in ROS inhibition in RAW 264.7 cells^11^. Theoretically, CBN is expected to ameliorate DOX-induced cardiac injury via inhibition of ROS production.

Silent information regulator 1 (Sirt1) is a well-known ROS regulator. Sirt1 has been widely reported to take part in DOX-induced oxidative stress and apoptosis^12^^,^ 13. Forkhead box transcription factor O1 (FOXO1) changes in response to cellular stimulation and maintains tissue homeostasis during the above-mentioned physiological and pathological processes. Substantial evidence indicates that function of FOXO1 depends on the modulation of downstream targets such as apoptosis- and anti-oxidative stress enzymes, and metabolic and immune regulators. The regulation of FOXO1 and its role might provide a significant avenue for the prevention and treatment of diseases^14^. As a regulator of Sirt1, FOXO1 has been reported to interacted with Sirt1 and to be involved in several biological process. Sirt1/FOXO1 signaling pathway has been verified to contribute to ROS production in several pathological stimulus^15^^-^ 17. Song et al. reported that Sirt1/FOXO1signaling involved in DOX-induced liver damage, and Dioscin, one natural product, exhibited protective effects against DOX-induced liver damage via suppression of oxidative stress, inflammation, and apoptosis through regulating Sirt1/FOXO1signaling^18^. However, whether CBN could inhibit DOX-induced ROS formation through Sirt1/FOXO1 signaling pathway still remains unverified.

Therefore, the present study aimed to explore the protective effects of CBN in DOX-induced cardiac injury. Our hypothesis was that CBN could inhibit DOX-induced cardiotoxicity by regulating Sirt1/FOXO1 signaling pathway.

## Methods

### Reagents

DOX was purchased from Sigma (#D1515, St. Louis, United States of America). CBN (purity, 99.85%) was purchased from MedChemExpress (#5058-13-9, New Jersey, United States of America). Ex-527 was purchased from Sigma (#E7034, St. Louis, United States of America) as well.

### Animals

Male 8-week-old C57BL/6 mice were purchased from the Chinese Academy of Medical Sciences and housed in plexiglass cages, with 12-h light/dark cycles at 22 ± 2°C, with free access to food and water. Mice were randomly assigned to four groups:

- Normal saline (NS, n = 10) with vehicle (NS-Vehicle, n = 10);
- NS with CBN (NS-CBN, n = 10);
- DOX with vehicle (DOX-Vehicle);
- DOX with CBN (DOX-CBN, n = 10).

DOX-induced cardiotoxicity was produced according to previous report^19^. Mice were treated with CBN (10 mg/kg/day, i.p.) four weeks after injection of DOX for four weeks to explore the potential role of CBN^9^. In addition, to further verify the role of Sirt1, a Sirt1 specific inhibitor (Ex-527, 1 mg/kg/day, i.p.) for one week at the end of CBN treatment^20^. All experimental procedures were according to the guidance of Guidelines for the Care and Use of Laboratory Animals published by the US National Institutes of Health and approved by the Animal Experiment Center of Wuhan Third Hospital (approval number: SY2022-001).

### Echocardiography analysis and hemodynamics

Transthoracic echocardiography was performed using a MyLab 30CV as described in previous studies^9^. Briefly, after inhalation anesthesia with 2% isoflurane, mice were placed in a shallow left lateral position. The left ventricular ejection fraction (LVEF) and short axis shortening rate (FS) were measured. Hemodynamic analysis was performed as previously reported^9^. Hemodynamic variables included +d*P*/dt and - d*P*/dt.

### Biochemical analysis

Serum levels of cardiac isoform of Tropnin T (cTnT), lactate dehydrogenase (LDH) and creatine kinase isoenzymes (CK-MB) were performed according to commercial kits according to manufacturer’s instructions^21^. Moreover, to detect myocardial oxidative injury, fresh heart samples were collected. The activities of superoxide dismutase (SOD), catalase (CAT), and the content of malondialdehyde (MDA) and 4-hydroxynonenal (4-HNE) by commercial kits were performed according to manufacturer’s instructions^21^.

### Reactive oxygen species measurement and TUNEL staining

To detect ROS, DHE staining was conducted. Fresh heart samples cryosections were prepared and stained with DHE for 30 min at room temperature. The images were photographed, and the fluorescence intensity of DHE was quantified by Image-Pro Plus software. TUNEL staining was performed using a commercial kit as previously described^21^.

### Quantitative real-time polymerase chain reaction and Western blot analysis

The total proteins were extracted from the frozen heart tissues. The quantitative real-time polymerase chain reaction (RT-qPCR) was conducted according to previously reported^21^. The sequences of the primers used for RT-qPCR are presented in Table 1. The mRNA levels were calculated using the relative standard curve method, and the calculated mRNA levels were normalized by glyceraldehyde-3-phosphate dehydrogenase (GAPDH) mRNA level.

**Table 1:** Mouse primers for real-time polymerase chain reaction.

Gene	Forward primers	Reverse primers
ANP	GGAGCAAATCCCGTATACAGTG	CTCTGAGACGGGTTGACTTCC
BNP	TCAAAGGACCAAGGCCCTAC	CTAAAACAACCTCAGCCCGTC
GAPDH	CGCTAACATCAAATGGGGTG	TTGCTGACAATCTTGAGGGAG

Western blotting was conducted as previously reported^21^. The primary antibody used in this manuscript were: Sirt1 (#9475, 1:1000, Cell Signaling Technology), Nrf2 (#ab137550, 1:500, Abcam), HO-1 (#43966, 1:1000, Cell Signaling Technology), Bcl2 (#ab196495, 1:500, Abcam), cle-caspase3 (#AF7022, 1:500, Affbiotech), Bax (#9475, 1:2000, Cell Signaling Technology), and GAPDH (#ab181602, 1:10000, Abcam).

### *Sirt1* deacetylase activity assay and immunoprecipitation of *FOXO1* acetylation

Sirt1 deacetylase activity was performed by using a commercial Sirt1 assay kit as previously described^22^. The immunoprecipitation of FOXO1 acetylation was conducted as previously described^18^. The primary antibodies used in this manuscript were anti-acetylated lysine (#AcK-103, Cell Signaling Technology) or anti-FOXO1 (#PA5-104560, ThermoFisher)^22^.

### Statistical analysis

All data were presented as mean ± standard error of the mean (SEM). Differences among three or more groups were compared using the one-way analysis of variance (ANOVA) analysis followed by Tukey’s test. *P* < 0.05 was considered statistically significant.

## Results

### *CBN* inhibited *DOX*-induced cardiac dysfunction in mice

**Figure 1:**
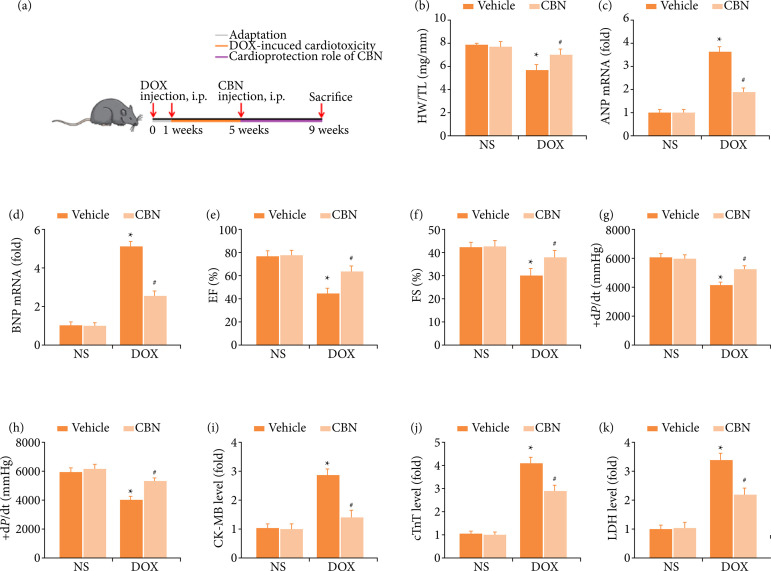
CBN inhibited DOX-induced cardiac dysfunction in mice. **(a)** Experimental protocol. **(b)** Statistical results of heart weight (HW)/tibia length (TL) (n = 10 per group). **(c and d)** Results of ANP and BNP mRNA levels (n = 6 per group). **(e and f)** Quantitative analysis of ejection fraction (EF), fraction shortening (FS) (n = 10 per group). **(g and h)** Quantitative analysis of +d*P*/dt and -d*P*/dt (n = 10 per group). **(i-k)** Statistical results of serum levels of CK-MB, cTnT and LDH (n = 10 per group).

CBN: columbianadin; DOX: doxorubicin; CK-MB: creatine kinase isoenzymes; cTnT: cardiac isoform of Tropnin T; LDH: lactate dehydrogenase; NS: normal saline; *P < 0.05 vs. NS-Vehicle group. #*P* < 0.05 vs. DOX-Vehicle group.

Figure 1a showed the study protocol. DOX treatment led to massive loss of cardiomyocytes, indicated by reduced heart weight (HW)/tibia length (TL) ratio (Fig. 1b). Previous study also demonstrated that DOX could induce cardiac hypertrophy. Our study verified DOX could increase cardiac hypertrophy, which exhibited by increased ANP and BNP mRNA expression (Figs. 1c and 1d). In addition, we found that DOX-induced an impaired cardiac function, which was exhibited by reduced EF, FS and ± d*P*/dt (Figs. 1e-1h).

Then, we next investigated whether the DOX administered caused cardiac injury. The results suggested that DOX markedly increased cardiac injury, which was shown as elevated CK-MB, cTnT and LDH (Figs. 1i-1k). Unsurprisingly, all above alterations induced by DOX treatment could be mitigated by CBN systematic administration. Collectively, we concluded that CBN could alleviate DOX-induced cardiotoxicity in mice.

### *CBN* decreased *DOX*-induced reactive oxygen species in hearts

**Figure 2:**
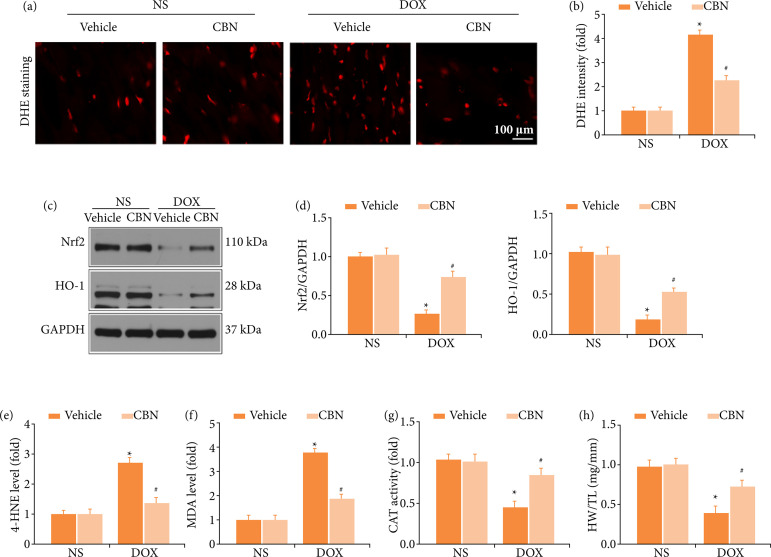
CBN decreased DOX-induced ROS in hearts. **(a)** Representative images and **(b)** quantitative results of DHE stained heart sections (n = 6 per group). **(c and d)** Representative Western blot and quantitative data (n = 4 per group). **(e-h)** The serum content of 4-HNE, MDA, CAT activity and SOD activity (n = 5 per group).

CBN: columbianadin; DOX: doxorubicin; ROS: reactive oxygen species; 4-HNE: 4-hydroxynonenal; MDA: malondialdehyde; CAT: catalase; SOD: superoxide dismutase; NS: normal saline; *P < 0.05 vs. NS-Vehicle group. #*P* < 0.05 vs. DOX-Vehicle group.

Oxidative stress was widely reported to be involved in the pathological mechanism of DOX-induced cardiotoxicity^23^. Then, we tested ROS generation in the heart. As depicted in Figs. 2a and 2b, DHE staining revealed that DOX treatment induced enhanced DHE fluorescence intensity in vivo and CBN administered significantly decreased ROS production. Western-blot results manifested that CBN up-regulated Bcl2 and HO-1 (Figs. 2c and 2d). Furthermore, we examined the serum biomarkers of ROS and found CBN curbed DOX-induced ROS over-production, which depicted by decreased serum 4-HNE, MDA levels and increased activity of CAT and SOD, compared to DOX-Vehicle group (Figs. 2e-2h). All these clues revealed an antioxidant effect of CBN against DOX-induced cardiac injury.

### *CBN* protected *DOX*-induced cardiomyocytes apoptosis in vivo

**Figure 3:**
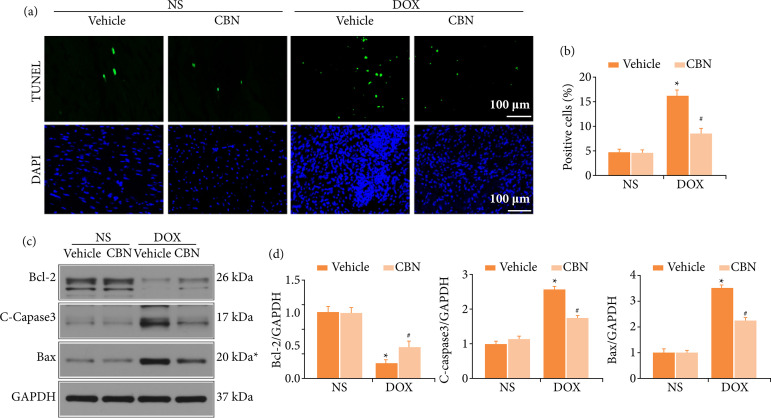
CBN protected DOX-induced cardiomyocytes apoptosis. **(a and b)** Representative TUNEL staining images and the quantitative results for apoptosis in heart tissues (n = 6 per group). **(c and d)** Representative Western blot and quantitative data (n = 4 per group).

CBN: columbianadin; DOX: doxorubicin; NS: normal saline; *P < 0.05 vs. NS-Vehicle group. #*P* < 0.05 vs. DOX-Vehicle group.

We then checked whether CBN could alleviate DOX-induced cardiomyocytes apoptosis. The TUNEL staining indicated that DOX treatment led to cell loss, and CBN administered markedly attenuated the alterations (Figs. 3a and 3b). This was further verified by western-blot results. CBN significantly reduced the protein levels of Bax and C-caspase3, elevated the Bcl-2 protein level following DOX administered (Figs. 3c and 3d).

### *Sirt1*/*FOXO1* signaling pathway contributes to the cardioprotective role of *CBN*

**Figure 4:**
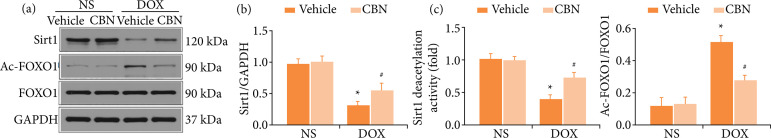
Sirt1/FOXO1 signaling pathway contributes to the protective role of CBN against DOX-induced ROS and apoptosis. **(a and b)** Representative Western blots, immunoprecipitation, and quantitative results of the Sirt1, Ac-FOXO1 and FOXO1 proteins (n = 4 per group). **(c)** Quantitative results of the Sirt1 deacetylation activity (n = 6 per group).

CBN: columbianadin; DOX: doxorubicin; NS: normal saline; *P < 0.05 vs. NS-Vehicle group. #*P* < 0.05 vs. DOX-Vehicle group; Sirt1: silent information regulator 1; FOXO1: forkhead box O1; ROS: reactive oxygen species.

Sirt1/FOXO1 signaling pathway was responsible for the ROS generation and cardiomyocytes apoptosis according to previously reported^24^. We then checked its role in this study. The results indicated that DOX down-regulated Sirt1 and up-regulated FOXO1, and CBN administered significantly increased Sirt1 and reduced FOXO1 protein expression (Figs. 4a and 4b). Sirt1 can deacetylate FOXO1 to exert its cardioprotection effect. We then tested Sirt1 deacetylation activity and found that DOX dampened Sirt1 deacetylation activity, and CBN treatment retained Sirt1 deacetylation activity (Fig. 4c).

### Inhibited *Sirt1* offset the cardioprotective effect of *CBN*

**Figure 5:**
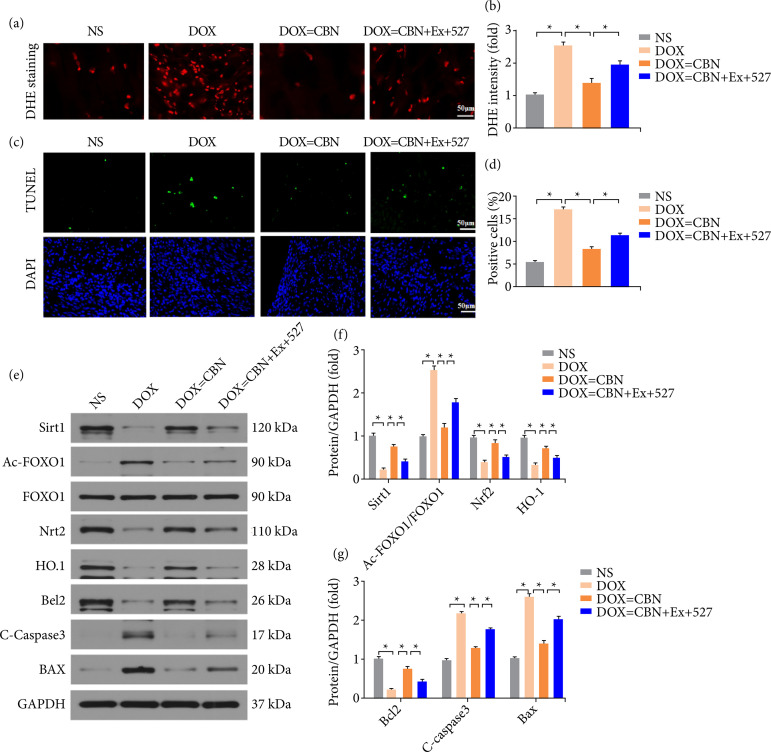
Inhibited Sirt1 partially reversed the beneficial effect of CBN on DOX-induced oxidative stress and apoptosis *in vivo*. **(a)** Representative images and **(b)** quantitative results of DHE stained heart sections (n = 6 per group). **(c and d)** Representative TUNEL staining images and the quantitative results for apoptosis in heart tissues (n = 6 per group). **(e and g)** Representative Western blot, immunoprecipitation, and quantitative data (n = 4 per group).

CBN: columbianadin; DOX: doxorubicin; *P < 0.05; Sirt1: silent information regulator 1.

To further confirm the role of Sirt1/FOXO1, we treated mice with Ex-527, a specific inhibitor of Sirt1. The results indicated that the decreased ROS over-production and cardiomyocytes apoptosis induced by CBN in DOX administered hearts were offset by Ex-527 (Figs. 5a-5d). Similar to the results of immunofluorescence staining, inhibitor Sirt1 with Ex-527 counteracted the antioxidant and anti-apoptosis effects of CBN, as indicated by the decreased protein expression of Nrf2 and HO-1, increased C-caspase3, Bax and decreased Bcl2 compared to DOX + CBN group (Figs. 5e-5g). Accordingly, we then checked the abolished role of Ex-527 in Sirt1/FOXO1 and found that Ex-527 treatment inactivated Sirt1/FOXO1 signaling, as demonstrated by decreased protein expression of Sirt1 and increased Ac-FOXO1 compared to DOX + CBN group (Fig. 5e and 5f).

### Inhibited *Sirt1* abolished the protective effect of *CBN* on *DOX*-induced cardiac dysfunction in hearts

**Figure 6:**
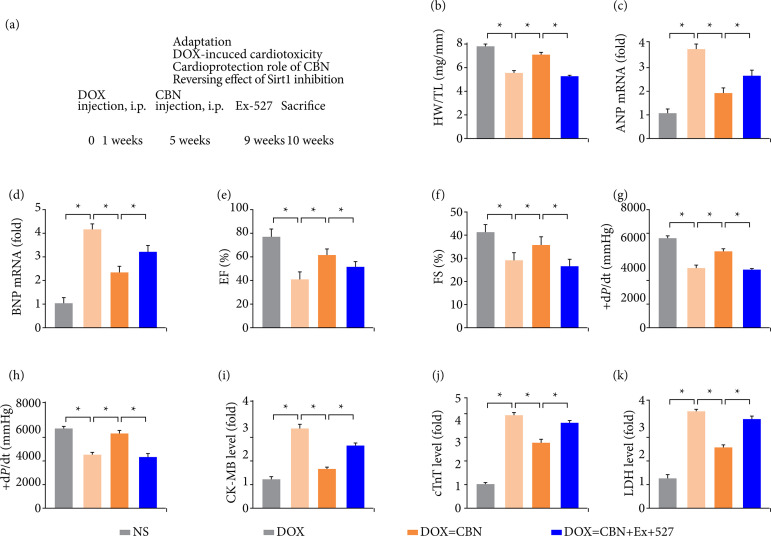
Inhibited Sirt1 partially abolished the protective effect of CBN on DOX-induced cardiac dysfunction. **(a)** Experimental protocol. **(b)** Statistical results of heart weight (HW)/tibia length (TL) (n = 10 per group). **(c and d)** Results of ANP and BNP mRNA levels (n = 6 per group). **(e and f)** Quantitative analysis of ejection fraction (EF) and fraction shortening (n = 10 per group). **(g and h)** Quantitative analysis of +dP/dt and -dP/dt (n = 10 per group). **(i-k)** Statistical results of serum levels of CK-MB, cTnT and LDH (n = 10 per group).

CBN: columbianadin; DOX: doxorubicin; *P < 0.05; Sirt1: silent information regulator 1; CK-MB: creatine kinase isoenzymes; cTnT: cardiac isoform of Tropnin T; LDH: lactate dehydrogenase.

Figure 6a showed the study protocol of the offset role of Sirt1 inhibition. DOX-treated mice following CBN treatment depicted elevated LVEF, Left ventricular fractional shortening (LVFS), ± d*P*/dt, HW/TL ratios reduced ANP, BNP mRNA (Figs. 6b-6h), and the improved cardiac function by CBN treatment were offset by Ex-527 administered (Figs. 6b-6h). Moreover, we also checked Sirt1 inhibition on biomarkers of cardiomyocyte damage. Our data indicated that CBN markedly decreased DOX-induced cardiomyocyte damage, which showed as reduced serum levels of CK-MB, cTnT and LDH, and these protective effects of CBN were reversed by Ex-527 administered (Figs. 6i-6k). Collectively, these results verified that CBN ameliorated DOX-induced cardiotoxicity by activating Sirt1/FOXO1 signaling pathway.

## Discussion

**Figure 7:**
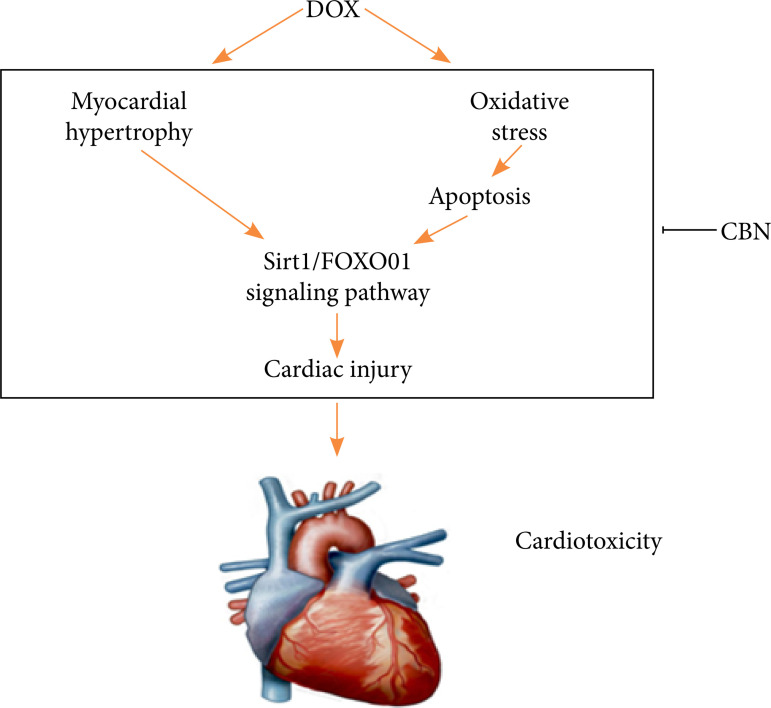
Schematic summary: mechanisms of CBN inhibited DOX-induced cardiotoxicity via regulating Sirt1/FOXO1 signaling pathway.

CBN: columbianadin; DOX: doxorubicin; Sirt1: silent information regulator 1; FOXO1: forkhead box O1.

DOX is an effectively therapy strategy clinically against several cancer. However, its clinical application was limited causing its side-effects, especially the cardiotoxicity, after long-term use, due to its highly affinity to the heart that eventually provokes cardiac injury and heart failure^2^. Hence, a promising pharmacological agent to curb this process is needed. Herein, we identified the cardioprotective role of CBN in DOX-related cardiac injury, cardiac dysfunction and elucidated the possible mechanisms. It is presumably associated with the antioxidant and anti-apoptotic actions of CBN. Mechanistically, we provided evidence that CBN activated Sirt1, decreased FOXO1 acetylation, and Ex-527 treated markedly reversed the protective role of CBN (Fig. 7).

DOX has been shown to induce cardiomyocyte damage through redox cycling and ROS production, which has been confirmed by multiple studies to be involved in one of the key processes in DOX-induced cardiomyocyte damage^25^. DOX-induced ROS over-production often due to a disturbed antioxidant defense system in the myocardium, which leads to a destruction of subcellular structure and activation of apoptotic cascades and finally causes heart failure^26^^,^ 27. In the present study, high levels of MDA, 4-HNE and low levels of SOD and CAT activities were found in myocardium treated with DOX.

Moreover, low protein expression of antioxidant indicators (Nrf2 and HO-1) was observed in DOX-induced myocardium, which was consistent with previous reported. Hu et al. also found high levels of MDA, 4-HNE and low levels of SOD and CAT activities in myocardium treated with DOX^21^. Targeting oxidative stress, previous studies indicated that several drugs including dexrazoxane, natural plant extracts, animal extracts, or chemically synthesized artificial antioxidants can decrease ROS formation, or elevate antioxidants, ultimately alleviate DOX-induced cardiac injury^4^^,^ 28. Among these agents, only dexrazoxane has been proven effective^29^. However, long-term and high-dose use will cause severe side effects.

Hence, another drug with effectiveness and safety needs to be explored. CBN is one of the main coumarin constituents isolated from *Angelica pubescens*, and it has been found to be involved in several diseases^7^^,^ 8. Previous studied mainly focused on inflammatory response. Zhang et al. aimed to investigate the anti-inflammatory effect of CBN on lipopolysaccharide (LPS)-stimulated THP-1 cells and found that CBN suppressed the LPS-mediated inflammatory response by inhibiting NOD1/NF-κB activation and concluded that CBN may be employed as a therapeutic agent for multiple inflammatory diseases^30^. However, whether CBN exhibited anti-oxidative stress effect remains unknown. In this paper, we uncovered an antioxidant role of CBN, which was exhibited by decreased serum levels of 4-HNE, MDA and increased activity of CAT and SOD. Moreover, in line with previously reported, we also proven that CBN played an anti-apoptotic role in this study^8^^,^ 30.

Another question is raised: how did CBN exhibit antioxidant and anti-apoptotic effects against DOX-induced cardiotoxicity? Many mechanisms have been explored to be involved in DOX-induced ROS and apoptosis. Hu et al. found that cAMP/PKA/Sirt1 pathway contributes to DOX-induced ROS and apoptosis^21^. Moreover, Liu et al. indicated that AMPK/PGC1α pathway activation may represent a new mechanism for melatonin exerted protection against acute DOX cardiotoxicity through alleviation of oxidative stress and apoptosis^31^.

In the present study, we intended to explored whether Sirt1/FOXO1 signaling involved in the cardioprotective effect of CBN in DOX-induced oxidative stress and apoptosis. Sirt1 is a NAD+ dependent histone deacetylase and plays indispensable roles in multiple pathophysiological processes, ranging from cell proliferation, differentiation, migration, aging to death. Previous study reported that Sirt1 serve as a sensing regulator involved in oxidative stress stimuli, and DOX could inhibit the activation of Sirt1, inhibited with Sirt1 aggravated DOX-induced cardiotoxicity^19^^,^ 32. Wang et al. demonstrated that FGF21 enhanced Nrf2 transcription activity and reduced cardiac ROS generation via Sirt1, while Sirt1 silence abolished this antioxidant effect^33^.

Moreover, Sirt1 activation could lead to p53 deacetylation and ultimately decreases cell apoptosis, whereas inhibiting Sirt1 augments p53 activity and aggravates apoptosis^34^. A recent study utilized a Sirt1 genetically modified mouse model and found that Sirt1 knockdown in heart (Sirt1f/f; MHCcre/+) mice by DOX treatment was more severe in cardiac dysfunction, apoptosis and DNA damage, and active Sirt1 with resveratrol could reverse DOX-related cardiac dysfunction^35^. Another study also manifested that both Sirt1 over-expression and Sirt1 agonist resveratrol can both reduced DOX-induced ROS production and cardiomyocyte apoptosis^36^. Similar to the previous results, our data indicated a reduced protein expression of Sirt1 following DOX administered. Meanwhile, activating Sirt1 by CBN could significantly alleviate DOX-induced cardiac injury, and Sirt1 inhibition counteracted the advantageous effects of CBN in DOX-induced cardiotoxicity, manifesting a cardioprotective role of CBN was mediated by Sirt1.

It is reported that Sirt1 exerts anti-ROS effect through acetylation with FOXO1^37^. Sirt1 has a NAD(+) binding domain and modulates the acetylation status of FOXO1. Sirt1 inhibits oxidative stress by causing deacetylation of FOXO1, enhancing its ability to bind to FOXO1 DNA^38^. Sin et al. provided evidence that DOX could decrease Sirt1 deacetylase activity, Sirt1 activator resveratrol could ameliorate the augmentation of pro-apoptotic markers including p53, Bax, and caspase 3 activity caused by DOX by decreasing DOX-induced increases in acetylation of FOXO1^39^.

Hence, we also intended to examine whether Sirt1/FOXO1 signaling linked to the beneficial role of CBN in this study. Our results showed that the protein expression and deacetylase activity of mice were significantly decreased after DOX stimulation, while CBN significantly enhanced the protein expression and deacetylase activity of Sirt1, resulting in a decrease in the acetylation level of FOXO1. The reversal experiment further demonstrated that inhibition of Sirt1 deacetylase activity with Ex-527 abolished the reversal effect of CBN on the DOX-induced increase in FOXO1 acetylation, thereby counteracting the protective effect of CBN on cardiac function.

However, there were some limitations in this manuscript. First, in this study, mice were treated with CBN four weeks after injection of DOX for four weeks to explore the potential role of CBN. However, the protective role of CBN was not evaluated at a shorter time. Meanwhile, CBN attenuated the effects of the chemotherapeutic drug DOX, whether CBN act on other chemotherapeutic agents has not been assessed.

## Conclusion

In summary, our study for the first time revealed that CBN treatment significantly ameliorated DOX-induced cardiotoxicity via oxidative stress and cardiomyocyte apoptosis. CBN administration caused Sirt1 activation and increased deacetylase activity of Sirt1, subsequently resulting in a decrease in the acetylation level of FOXO1 to restrain ROS over-formation and apoptosis. Our results indicated CBN as a promising therapeutic agent for the treatment of DOX-induced cardiac injury.

## Data Availability

The analyzed datasets generated in the present study are available from the corresponding author upon reasonable request.
